# 2-[(*E*)-(5-Amino-2,3-diphenyl­quinoxalin-6-yl)imino­meth­yl]-4-chloro­phenol

**DOI:** 10.1107/S1600536808020023

**Published:** 2008-07-05

**Authors:** Hoong-Kun Fun, Reza Kia, Paul R. Raithby

**Affiliations:** a X-ray Crystallography Unit, School of Physics, Universiti Sains Malaysia, 11800 USM, Penang, Malaysia; b Chemistry Department, University of Bath, Claverton Down, Bath BA2 7AY, England

## Abstract

The title Schiff base compound, C_27_H_19_ClN_4_O, features two intra­molecular O—H⋯N and N—H⋯N hydrogen bonds involving the hydr­oxy and amino groups to generate *S*(6) and *S*(5) ring motifs, respectively. In the crystal structure, weak inter­molecular N—H⋯O and C—H⋯N inter­actions, together with π–π contacts [centroid–centroid distances = 3.6294 (11)–3.6881 (11) Å], link neighboring mol­ecules.

## Related literature

For related literature on hydrogen-bond motifs, see: Bernstein *et al.* (1995 ▶). For related structures, see: Corden *et al.* (1996 ▶); Fun *et al.* (2008 ▶); Govindasamy *et al.* (1999 ▶). For applications and bioactivities, see: Anderson *et al.* (1997 ▶); Cohen & Schmidt (1964 ▶); Granovski *et al.* (1993 ▶); Li & Chang (1991 ▶); Shahrokhian *et al.* (2000 ▶); Unaleroglu & Hökelek (2002 ▶).
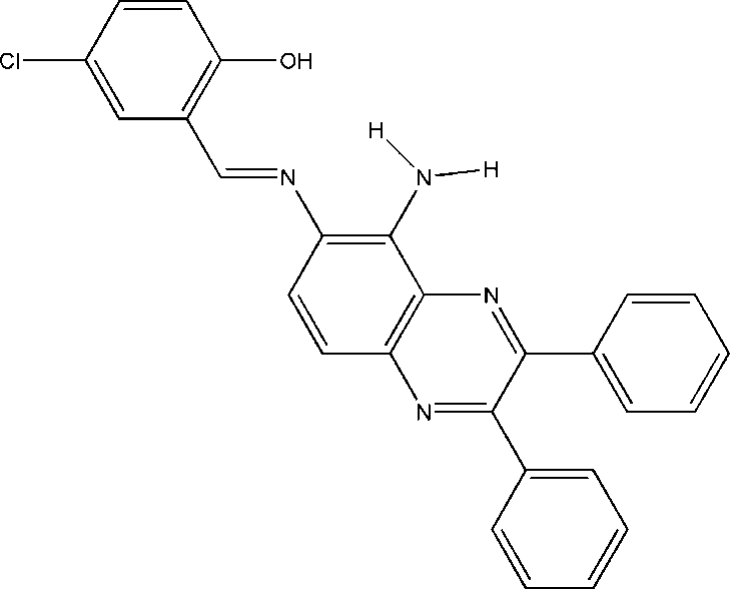


## Experimental

### 

#### Crystal data

C_27_H_19_ClN_4_O
*M* *_r_* = 450.91
Monoclinic, 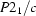

*a* = 22.8728 (11) Å
*b* = 7.3068 (4) Å
*c* = 12.5632 (6) Å
β = 92.037 (2)°
*V* = 2098.32 (18) Å^3^
*Z* = 4
Mo *K*α radiation
μ = 0.21 mm^−1^
*T* = 100.0 (1) K
0.55 × 0.09 × 0.07 mm

#### Data collection

Bruker SMART APEXII CCD area-detector diffractometer
Absorption correction: multi-scan (*SADABS*; Bruker, 2005 ▶) *T*_min_ = 0.843, *T*_max_ = 0.985
25593 measured reflections
6180 independent reflections
4118 reflections with *I* > 2σ(*I*)
*R*_int_ = 0.062

#### Refinement

*R*[*F* ^2^ > 2σ(*F* ^2^)] = 0.052
*wR*(*F* ^2^) = 0.159
*S* = 1.01
6180 reflections
310 parameters
H atoms treated by a mixture of independent and constrained refinement
Δρ_max_ = 0.40 e Å^−3^
Δρ_min_ = −0.41 e Å^−3^

### 

Data collection: *APEX2* (Bruker, 2005 ▶); cell refinement: *APEX2*; data reduction: *SAINT* (Bruker, 2005 ▶); program(s) used to solve structure: *SHELXTL* (Sheldrick, 2008 ▶); program(s) used to refine structure: *SHELXTL*; molecular graphics: *SHELXTL*; software used to prepare material for publication: *SHELXTL* and *PLATON* (Spek, 2003 ▶).

## Supplementary Material

Crystal structure: contains datablocks global, I. DOI: 10.1107/S1600536808020023/tk2279sup1.cif

Structure factors: contains datablocks I. DOI: 10.1107/S1600536808020023/tk2279Isup2.hkl

Additional supplementary materials:  crystallographic information; 3D view; checkCIF report

## Figures and Tables

**Table 1:** Hydrogen-bond geometry (Å, °)

*D*—H⋯*A*	*D*—H	H⋯*A*	*D*⋯*A*	*D*—H⋯*A*
O1—H1O1⋯N1	0.93 (3)	1.72 (3)	2.584 (2)	153 (2)
N4—H1N4⋯O1^i^	1.01 (3)	2.47 (3)	3.099 (2)	120.4 (18)
N4—H2N4⋯N2	1.04 (3)	2.31 (3)	2.750 (2)	104.3 (17)
C27—H27*A*⋯N3^ii^	0.93	2.62	3.373 (2)	138

Symmetry codes: (i) 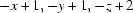; (ii) 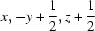.

**Fig. 1.**
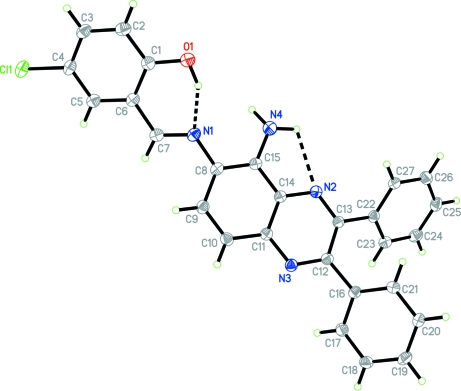
The molecular structure of (I), showing 50% probability displacement ellipsoids and atomic numbering. Intramolecular interactions are drawn as dashed lines.

**Fig. 2.**
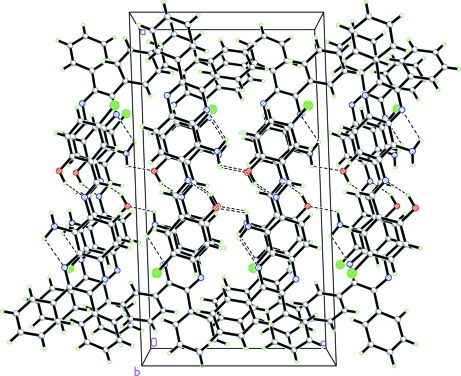
The crystal packing in (I), viewed down the b-axis showing stacking arrangement. Intramolecular and intermolecular interactions are shown as dashed lines.

## supplementary crystallographic information

### Comment

Schiff bases are one of the most prevalent mixed-donor ligand types in coordination chemistry. The biologically activities of their complexes have been studied extensively over the last decades (Anderson *et al.*, 1997; Corden *et al.,* 1996; Govindasamy *et al.*, 1999; Granovski *et al.*, 1993; Li & Chang, 1991; Shahrokhian *et al.*, 2000). 2-Hydroxy Schiff base ligands are of additional interest mainly due to the existence of O—H···N and O···H—N type hydrogen bonds and tautomerization between the phenol-imine and keto-amine forms (Unaleroglu & Hokelek, 2002; Fun *et al.*, 2008). This type of tautomerism plays an important role for distinguishing their photochromic and thermochromic properties (Cohen & Schmidt, 1964). Knowing the solution and solid-state structures of free Schiff bases is important in view of the intramolecular hydrogen bonding and comparing conformation with that in the structures of Schiff base complexes. In view of the importance of these organic ligands, the title compound (I) was synthesized and its crystal structure is reported herein.

Compound (I, Fig. 1), features intramolecular O—H···N and N—H···N hydrogen bonds to form six- and five-membered rings, producing S(6) and S(5) ring motifs, respectively (Bernstein *et al.*, 1995). The two phenyl substituents on the quinoxaline unit are inclined at an angle of 61.14 (9)° to one another. They also form dihedral angles of 43.38 (9) and 39.50 (9)°, respectively, with the ten–membered quinoxaline ring. In the crystal packing (Table 1 & Fig. 2), molecules are stacked when viewed down the *b* axis, being consolidated by π–π interactions with *Cg2*···*Cg3* distances ranging from 3.6294 (11) – 3.6881 (11) Å; symmetry codes 1 - *x*, -1/2 + *y*, 3/2 - *z* and 1 - *x*, 1/2 + *y*, 3/2 - *z*, and *Cg2* and *Cg3* are the centroids of the C1–C6 and C8/C9/C10/C11/C14/C15 phenyl rings, respectively. The crystal structure is also stabilized by intramolecular O—H···N and N—H···N contacts.

### Experimental

The synthetic method used for the preparation of (I) has been described earlier (Fun *et al.*, 2008). Single crystals suitable for *X*-ray diffraction were obtained by evaporation of a mixed dichloromethane-ethanol (3/1) solution of (I), held at room temperature.

### Refinement

The H-atoms attached to O1 and N4 were located in a difference Fourier map and refined freely; see Table 1 for bond distances. The remaining H atoms were included in the riding model approximation with C—H = 0.93 Å, and with U_iso_(H) = 1.2*U*_eq_(C).

### Crystal data

**Table:**

C_27_H_19_ClN_4_O	*F*_000_ = 936
*M**_r_* = 450.91	*D*_x_ = 1.427 Mg m^−^^3^
Monoclinic, *P*2_1_/*c*	Mo *K*α radiation λ = 0.71073 Å
Hall symbol: -P 2ybc	Cell parameters from 2969 reflections
*a* = 22.8728 (11) Å	θ = 2.9–28.1º
*b* = 7.3068 (4) Å	µ = 0.21 mm^−^^1^
*c* = 12.5632 (6) Å	*T* = 100.0 (1) K
β = 92.037 (2)º	Block, yellow
*V* = 2098.32 (18) Å^3^	0.55 × 0.09 × 0.07 mm
*Z* = 4	

### Data collection

**Table:**

Bruker SMART APEXII CCD area-detector diffractometer	6180 independent reflections
Radiation source: fine-focus sealed tube	4118 reflections with *I* > 2σ(*I*)
Monochromator: graphite	*R*_int_ = 0.062
*T* = 100.0(1) K	θ_max_ = 30.1º
φ and ω scans	θ_min_ = 0.9º
Absorption correction: multi-scan(SADABS; Bruker, 2005)	*h* = −32→31
*T*_min_ = 0.843, *T*_max_ = 0.985	*k* = −10→10
25593 measured reflections	*l* = −17→17

### Refinement

**Table:**

Refinement on *F*^2^	Secondary atom site location: difference Fourier map
Least-squares matrix: full	Hydrogen site location: inferred from neighbouring sites
*R*[*F*^2^ > 2σ(*F*^2^)] = 0.052	H atoms treated by a mixture of independent and constrained refinement
*wR*(*F*^2^) = 0.159	*w* = 1/[σ^2^(*F*_o_^2^) + (0.0879*P*)^2^] where *P* = (*F*_o_^2^ + 2*F*_c_^2^)/3
*S* = 1.01	(Δ/σ)_max_ = 0.001
6180 reflections	Δρ_max_ = 0.40 e Å^−^^3^
310 parameters	Δρ_min_ = −0.41 e Å^−^^3^
Primary atom site location: structure-invariant direct methods	Extinction correction: none

### Special details

**Table:**

Experimental. The low-temperature data was collected with the Oxford Cyrosystem Cobra low-temperature attachment.
Geometry. All e.s.d.'s (except the e.s.d. in the dihedral angle between two l.s. planes) are estimated using the full covariance matrix. The cell e.s.d.'s are taken into account individually in the estimation of e.s.d.'s in distances, angles and torsion angles; correlations between e.s.d.'s in cell parameters are only used when they are defined by crystal symmetry. An approximate (isotropic) treatment of cell e.s.d.'s is used for estimating e.s.d.'s involving l.s. planes.
Refinement. Refinement of *F*^2^ against ALL reflections. The weighted *R*-factor *wR* and goodness of fit *S* are based on *F*^2^, conventional *R*-factors *R* are based on *F*, with *F* set to zero for negative *F*^2^. The threshold expression of *F*^2^ > σ(*F*^2^) is used only for calculating *R*-factors(gt) *etc*. and is not relevant to the choice of reflections for refinement. *R*-factors based on *F*^2^ are statistically about twice as large as those based on *F*, and *R*- factors based on ALL data will be even larger.

### Fractional atomic coordinates and isotropic or equivalent isotropic displacement parameters (Å^2^)

**Table:**

	*x*	*y*	*z*	*U*_iso_*/*U*_eq_	
Cl1	0.26328 (2)	0.46708 (7)	0.59559 (4)	0.02813 (14)	
O1	0.44768 (6)	0.2687 (2)	0.91204 (10)	0.0252 (3)	
N1	0.52358 (6)	0.3641 (2)	0.77495 (12)	0.0200 (3)	
N2	0.72297 (7)	0.4713 (2)	0.88154 (11)	0.0174 (3)	
N3	0.75971 (6)	0.3333 (2)	0.68619 (11)	0.0185 (3)	
N4	0.60625 (8)	0.4640 (2)	0.92698 (13)	0.0263 (4)	
C1	0.40599 (8)	0.3116 (3)	0.83649 (14)	0.0203 (4)	
C2	0.34744 (8)	0.2879 (3)	0.85952 (15)	0.0236 (4)	
H2A	0.3375	0.2402	0.9251	0.028*	
C3	0.30418 (8)	0.3350 (3)	0.78542 (15)	0.0233 (4)	
H3A	0.2651	0.3199	0.8013	0.028*	
C4	0.31874 (8)	0.4049 (3)	0.68704 (15)	0.0214 (4)	
C5	0.37624 (8)	0.4249 (3)	0.66116 (14)	0.0211 (4)	
H5A	0.3855	0.4700	0.5946	0.025*	
C6	0.42106 (8)	0.3773 (2)	0.73536 (14)	0.0197 (4)	
C7	0.48149 (8)	0.3948 (3)	0.70633 (14)	0.0206 (4)	
H7A	0.4901	0.4287	0.6373	0.025*	
C8	0.58278 (7)	0.3677 (2)	0.74723 (14)	0.0183 (3)	
C9	0.60160 (8)	0.3128 (3)	0.64598 (14)	0.0206 (4)	
H9A	0.5739	0.2813	0.5932	0.025*	
C10	0.65962 (8)	0.3050 (3)	0.62430 (14)	0.0205 (4)	
H10A	0.6713	0.2684	0.5574	0.025*	
C11	0.70168 (7)	0.3530 (2)	0.70445 (13)	0.0179 (3)	
C12	0.79775 (7)	0.3790 (2)	0.76358 (13)	0.0175 (3)	
C13	0.77919 (7)	0.4620 (2)	0.86047 (13)	0.0169 (3)	
C14	0.68362 (7)	0.4111 (2)	0.80547 (13)	0.0169 (3)	
C15	0.62300 (8)	0.4161 (2)	0.82744 (13)	0.0173 (3)	
C16	0.85933 (7)	0.3244 (2)	0.74618 (13)	0.0186 (4)	
C17	0.88232 (8)	0.3426 (3)	0.64549 (14)	0.0211 (4)	
H17A	0.8603	0.3993	0.5913	0.025*	
C18	0.93766 (8)	0.2771 (3)	0.62536 (14)	0.0232 (4)	
H18A	0.9529	0.2925	0.5583	0.028*	
C19	0.97043 (8)	0.1884 (3)	0.70519 (15)	0.0241 (4)	
H19A	1.0072	0.1419	0.6912	0.029*	
C20	0.94797 (8)	0.1695 (3)	0.80613 (14)	0.0221 (4)	
H20A	0.9698	0.1112	0.8600	0.027*	
C21	0.89277 (8)	0.2380 (3)	0.82614 (13)	0.0217 (4)	
H21A	0.8780	0.2260	0.8938	0.026*	
C22	0.82037 (8)	0.5458 (2)	0.93956 (13)	0.0181 (4)	
C23	0.86747 (8)	0.6521 (3)	0.90850 (14)	0.0218 (4)	
H23A	0.8743	0.6675	0.8365	0.026*	
C24	0.90422 (8)	0.7350 (3)	0.98433 (15)	0.0250 (4)	
H24A	0.9350	0.8081	0.9630	0.030*	
C25	0.89514 (8)	0.7091 (3)	1.09180 (15)	0.0244 (4)	
H25A	0.9205	0.7617	1.1426	0.029*	
C26	0.84845 (8)	0.6051 (3)	1.12332 (14)	0.0227 (4)	
H26A	0.8422	0.5887	1.1954	0.027*	
C27	0.81078 (8)	0.5246 (2)	1.04769 (13)	0.0194 (4)	
H27A	0.7790	0.4563	1.0694	0.023*	
H1O1	0.4828 (12)	0.299 (4)	0.881 (2)	0.062 (9)*	
H1N4	0.5681 (12)	0.531 (4)	0.9337 (19)	0.052 (8)*	
H2N4	0.6396 (13)	0.516 (4)	0.976 (2)	0.060 (8)*	

### Atomic displacement parameters (Å^2^)

**Table:**

	*U*^11^	*U*^22^	*U*^33^	*U*^12^	*U*^13^	*U*^23^
Cl1	0.0190 (2)	0.0284 (3)	0.0367 (3)	0.0003 (2)	−0.00334 (18)	−0.0011 (2)
O1	0.0226 (7)	0.0291 (8)	0.0241 (6)	0.0010 (6)	0.0023 (5)	0.0007 (6)
N1	0.0176 (7)	0.0168 (7)	0.0256 (8)	−0.0017 (6)	0.0014 (6)	−0.0025 (6)
N2	0.0188 (7)	0.0151 (7)	0.0184 (7)	0.0010 (6)	0.0009 (5)	0.0010 (6)
N3	0.0183 (7)	0.0180 (7)	0.0194 (7)	0.0015 (6)	0.0001 (5)	0.0003 (6)
N4	0.0242 (8)	0.0304 (9)	0.0242 (8)	0.0028 (8)	0.0009 (6)	−0.0002 (7)
C1	0.0223 (9)	0.0162 (8)	0.0223 (8)	−0.0010 (7)	0.0013 (7)	−0.0036 (7)
C2	0.0245 (9)	0.0206 (9)	0.0262 (9)	−0.0020 (8)	0.0060 (7)	−0.0033 (7)
C3	0.0182 (8)	0.0210 (9)	0.0308 (9)	−0.0009 (8)	0.0039 (7)	−0.0060 (8)
C4	0.0178 (8)	0.0161 (8)	0.0302 (9)	0.0003 (7)	−0.0017 (7)	−0.0042 (7)
C5	0.0212 (9)	0.0173 (9)	0.0251 (9)	−0.0020 (8)	0.0022 (7)	−0.0012 (7)
C6	0.0191 (8)	0.0158 (8)	0.0244 (9)	−0.0004 (7)	0.0017 (7)	−0.0026 (7)
C7	0.0212 (9)	0.0174 (9)	0.0234 (8)	−0.0006 (8)	0.0027 (7)	−0.0012 (7)
C8	0.0170 (8)	0.0142 (8)	0.0237 (8)	−0.0002 (7)	0.0021 (6)	0.0005 (7)
C9	0.0196 (8)	0.0191 (9)	0.0230 (8)	−0.0005 (7)	−0.0012 (7)	−0.0031 (7)
C10	0.0225 (9)	0.0197 (9)	0.0193 (8)	0.0021 (8)	0.0002 (7)	−0.0018 (7)
C11	0.0178 (8)	0.0164 (8)	0.0194 (8)	0.0004 (7)	0.0007 (6)	−0.0009 (7)
C12	0.0181 (8)	0.0164 (8)	0.0179 (8)	0.0002 (7)	0.0022 (6)	0.0024 (6)
C13	0.0168 (8)	0.0161 (8)	0.0179 (8)	0.0007 (7)	0.0011 (6)	0.0019 (6)
C14	0.0176 (8)	0.0142 (8)	0.0190 (8)	0.0011 (7)	0.0014 (6)	0.0012 (6)
C15	0.0185 (8)	0.0135 (8)	0.0199 (8)	0.0017 (7)	0.0024 (6)	0.0016 (7)
C16	0.0164 (8)	0.0178 (8)	0.0218 (8)	−0.0005 (7)	0.0009 (6)	−0.0016 (7)
C17	0.0198 (8)	0.0230 (9)	0.0206 (8)	−0.0022 (8)	0.0006 (7)	0.0013 (7)
C18	0.0200 (9)	0.0296 (10)	0.0202 (8)	−0.0031 (8)	0.0032 (7)	−0.0018 (7)
C19	0.0154 (8)	0.0278 (10)	0.0292 (9)	0.0005 (8)	0.0034 (7)	−0.0014 (8)
C20	0.0178 (8)	0.0251 (10)	0.0234 (9)	0.0030 (8)	−0.0014 (7)	0.0012 (8)
C21	0.0212 (9)	0.0260 (10)	0.0182 (8)	0.0013 (8)	0.0030 (7)	−0.0001 (7)
C22	0.0171 (8)	0.0165 (8)	0.0204 (8)	0.0032 (7)	−0.0008 (6)	−0.0015 (7)
C23	0.0205 (8)	0.0220 (9)	0.0231 (8)	0.0011 (8)	0.0032 (7)	−0.0005 (7)
C24	0.0200 (9)	0.0237 (10)	0.0314 (10)	−0.0031 (8)	0.0035 (7)	−0.0025 (8)
C25	0.0198 (9)	0.0250 (10)	0.0280 (9)	0.0008 (8)	−0.0040 (7)	−0.0052 (8)
C26	0.0254 (9)	0.0224 (9)	0.0202 (8)	0.0025 (8)	−0.0003 (7)	−0.0006 (7)
C27	0.0170 (8)	0.0202 (9)	0.0210 (8)	−0.0001 (7)	0.0023 (6)	−0.0011 (7)

### Geometric parameters (Å, °)

**Table:**

Cl1—C4	1.7414 (19)	C10—H10A	0.9300
O1—C1	1.358 (2)	C11—C14	1.414 (2)
O1—H1O1	0.93 (3)	C12—C13	1.437 (2)
N1—C7	1.289 (2)	C12—C16	1.488 (2)
N1—C8	1.410 (2)	C13—C22	1.478 (2)
N2—C13	1.324 (2)	C14—C15	1.424 (2)
N2—C14	1.362 (2)	C16—C21	1.392 (2)
N3—C12	1.324 (2)	C16—C17	1.393 (2)
N3—C11	1.363 (2)	C17—C18	1.385 (2)
N4—C15	1.366 (2)	C17—H17A	0.9300
N4—H1N4	1.01 (3)	C18—C19	1.391 (3)
N4—H2N4	1.03 (3)	C18—H18A	0.9300
C1—C2	1.391 (3)	C19—C20	1.392 (3)
C1—C6	1.412 (2)	C19—H19A	0.9300
C2—C3	1.378 (3)	C20—C21	1.389 (2)
C2—H2A	0.9300	C20—H20A	0.9300
C3—C4	1.389 (3)	C21—H21A	0.9300
C3—H3A	0.9300	C22—C27	1.392 (2)
C4—C5	1.374 (2)	C22—C23	1.395 (2)
C5—C6	1.405 (2)	C23—C24	1.387 (3)
C5—H5A	0.9300	C23—H23A	0.9300
C6—C7	1.448 (2)	C24—C25	1.386 (3)
C7—H7A	0.9300	C24—H24A	0.9300
C8—C15	1.386 (2)	C25—C26	1.380 (3)
C8—C9	1.415 (2)	C25—H25A	0.9300
C9—C10	1.365 (2)	C26—C27	1.390 (2)
C9—H9A	0.9300	C26—H26A	0.9300
C10—C11	1.412 (2)	C27—H27A	0.9300
			
C1—O1—H1O1	104.0 (16)	N2—C13—C22	116.42 (15)
C7—N1—C8	122.17 (15)	C12—C13—C22	122.95 (15)
C13—N2—C14	117.74 (14)	N2—C14—C11	121.32 (15)
C12—N3—C11	117.91 (15)	N2—C14—C15	118.65 (15)
C15—N4—H1N4	118.3 (14)	C11—C14—C15	119.96 (16)
C15—N4—H2N4	114.3 (16)	N4—C15—C8	122.02 (16)
H1N4—N4—H2N4	113 (2)	N4—C15—C14	119.38 (16)
O1—C1—C2	118.90 (16)	C8—C15—C14	118.58 (15)
O1—C1—C6	121.29 (16)	C21—C16—C17	118.89 (16)
C2—C1—C6	119.81 (17)	C21—C16—C12	120.97 (15)
C3—C2—C1	120.12 (17)	C17—C16—C12	119.83 (15)
C3—C2—H2A	119.9	C18—C17—C16	120.63 (17)
C1—C2—H2A	119.9	C18—C17—H17A	119.7
C2—C3—C4	120.27 (17)	C16—C17—H17A	119.7
C2—C3—H3A	119.9	C17—C18—C19	120.12 (16)
C4—C3—H3A	119.9	C17—C18—H18A	119.9
C5—C4—C3	120.78 (17)	C19—C18—H18A	119.9
C5—C4—Cl1	119.81 (14)	C18—C19—C20	119.77 (17)
C3—C4—Cl1	119.40 (14)	C18—C19—H19A	120.1
C4—C5—C6	119.91 (17)	C20—C19—H19A	120.1
C4—C5—H5A	120.0	C21—C20—C19	119.75 (17)
C6—C5—H5A	120.0	C21—C20—H20A	120.1
C5—C6—C1	119.04 (16)	C19—C20—H20A	120.1
C5—C6—C7	119.45 (16)	C20—C21—C16	120.81 (16)
C1—C6—C7	121.51 (16)	C20—C21—H21A	119.6
N1—C7—C6	120.88 (16)	C16—C21—H21A	119.6
N1—C7—H7A	119.6	C27—C22—C23	119.01 (16)
C6—C7—H7A	119.6	C27—C22—C13	119.40 (16)
C15—C8—N1	116.33 (15)	C23—C22—C13	121.53 (15)
C15—C8—C9	120.70 (16)	C24—C23—C22	120.41 (16)
N1—C8—C9	122.82 (15)	C24—C23—H23A	119.8
C10—C9—C8	121.32 (16)	C22—C23—H23A	119.8
C10—C9—H9A	119.3	C25—C24—C23	120.07 (18)
C8—C9—H9A	119.3	C25—C24—H24A	120.0
C9—C10—C11	119.34 (16)	C23—C24—H24A	120.0
C9—C10—H10A	120.3	C26—C25—C24	119.92 (17)
C11—C10—H10A	120.3	C26—C25—H25A	120.0
N3—C11—C10	119.78 (15)	C24—C25—H25A	120.0
N3—C11—C14	120.02 (15)	C25—C26—C27	120.26 (16)
C10—C11—C14	120.09 (16)	C25—C26—H26A	119.9
N3—C12—C13	121.47 (15)	C27—C26—H26A	119.9
N3—C12—C16	115.16 (15)	C26—C27—C22	120.28 (17)
C13—C12—C16	123.18 (15)	C26—C27—H27A	119.9
N2—C13—C12	120.61 (15)	C22—C27—H27A	119.9
			
O1—C1—C2—C3	178.24 (16)	C10—C11—C14—N2	−175.02 (16)
C6—C1—C2—C3	−2.5 (3)	N3—C11—C14—C15	−174.42 (16)
C1—C2—C3—C4	0.5 (3)	C10—C11—C14—C15	1.8 (3)
C2—C3—C4—C5	1.3 (3)	N1—C8—C15—N4	−2.2 (3)
C2—C3—C4—Cl1	−178.91 (14)	C9—C8—C15—N4	−177.84 (17)
C3—C4—C5—C6	−1.1 (3)	N1—C8—C15—C14	176.25 (16)
Cl1—C4—C5—C6	179.12 (14)	C9—C8—C15—C14	0.6 (3)
C4—C5—C6—C1	−0.9 (3)	N2—C14—C15—N4	−6.2 (3)
C4—C5—C6—C7	178.53 (17)	C11—C14—C15—N4	176.85 (17)
O1—C1—C6—C5	−178.08 (16)	N2—C14—C15—C8	175.28 (16)
C2—C1—C6—C5	2.7 (3)	C11—C14—C15—C8	−1.6 (3)
O1—C1—C6—C7	2.5 (3)	N3—C12—C16—C21	−132.71 (18)
C2—C1—C6—C7	−176.75 (17)	C13—C12—C16—C21	42.4 (3)
C8—N1—C7—C6	175.52 (16)	N3—C12—C16—C17	40.8 (2)
C5—C6—C7—N1	175.24 (17)	C13—C12—C16—C17	−144.14 (18)
C1—C6—C7—N1	−5.3 (3)	C21—C16—C17—C18	−0.4 (3)
C7—N1—C8—C15	151.46 (17)	C12—C16—C17—C18	−174.06 (17)
C7—N1—C8—C9	−33.0 (3)	C16—C17—C18—C19	1.4 (3)
C15—C8—C9—C10	0.3 (3)	C17—C18—C19—C20	−1.5 (3)
N1—C8—C9—C10	−175.08 (17)	C18—C19—C20—C21	0.5 (3)
C8—C9—C10—C11	−0.1 (3)	C19—C20—C21—C16	0.5 (3)
C12—N3—C11—C10	−179.68 (16)	C17—C16—C21—C20	−0.6 (3)
C12—N3—C11—C14	−3.4 (3)	C12—C16—C21—C20	173.01 (17)
C9—C10—C11—N3	175.32 (17)	N2—C13—C22—C27	40.2 (2)
C9—C10—C11—C14	−0.9 (3)	C12—C13—C22—C27	−141.69 (18)
C11—N3—C12—C13	−5.4 (3)	N2—C13—C22—C23	−137.21 (17)
C11—N3—C12—C16	169.78 (15)	C12—C13—C22—C23	40.9 (3)
C14—N2—C13—C12	−4.3 (2)	C27—C22—C23—C24	0.1 (3)
C14—N2—C13—C22	173.81 (15)	C13—C22—C23—C24	177.55 (17)
N3—C12—C13—N2	9.7 (3)	C22—C23—C24—C25	1.5 (3)
C16—C12—C13—N2	−165.05 (17)	C23—C24—C25—C26	−1.9 (3)
N3—C12—C13—C22	−168.31 (16)	C24—C25—C26—C27	0.7 (3)
C16—C12—C13—C22	16.9 (3)	C25—C26—C27—C22	1.0 (3)
C13—N2—C14—C11	−4.5 (3)	C23—C22—C27—C26	−1.4 (3)
C13—N2—C14—C15	178.63 (16)	C13—C22—C27—C26	−178.89 (17)
N3—C11—C14—N2	8.7 (3)		

### Hydrogen-bond geometry (Å, °)

**Table:**

*D*—H···*A*	*D*—H	H···*A*	*D*···*A*	*D*—H···*A*
O1—H1O1···N1	0.93 (3)	1.72 (3)	2.584 (2)	153 (2)
N4—H1N4···O1^i^	1.01 (3)	2.47 (3)	3.099 (2)	120.4 (18)
N4—H2N4···N2	1.04 (3)	2.31 (3)	2.750 (2)	104.3 (17)
C27—H27A···N3^ii^	0.93	2.62	3.373 (2)	138

Symmetry codes: (i) −*x*+1, −*y*+1, −*z*+2; (ii) *x*, −*y*+1/2, *z*+1/2.
